# 25-Hydroxyvitamin D Levels and the Risk of Dementia and Alzheimer's Disease: A Dose–Response Meta-Analysis

**DOI:** 10.3389/fnagi.2018.00368

**Published:** 2018-11-09

**Authors:** Hanze Chen, Weishuang Xue, Jinwei Li, Kailei Fu, Han Shi, Beidi Zhang, Weiyu Teng, Li Tian

**Affiliations:** ^1^Deparment of Neurology, The First Affiliated Hospital, China Medical University, Shenyang, China; ^2^Clinical Department one, China Medical University, Shenyang, China; ^3^Department of Endodontics, School of Stomatology, China Medical University, Shenyang, China; ^4^Deparment of Geriatrics, Shengjing Hospital, China Medical University, Shenyang, China

**Keywords:** dementia, Alzheimer's disease, 25-hydroxyvitamin D, meta-analysis, cohort studies

## Abstract

**Background and Purpose:** Conclusions of previous cohort studies on the relationship between 25-hydroxyvitamin D level and the risk of dementia and Alzheimer's disease were not consistent. Thus, we performed a dose–response meta-analysis to evaluate this relationship by summarizing cohort studies.

**Methods:** Pubmed, Embase, Cochrane, and Web of Science databases were searched for relevant studies. Cohort studies concerning the association between 25-hydroxyvitamin D level and dementia or Alzheimer's disease were included. Results of studies were pooled and the dose–response relationship was determined using a random-effect model.

**Results:** Ten cohort studies, with 28,640 participants were included. A significant inverse relationship was found between 25-hydroxyvitamin D level and the risk of dementia and Alzheimer's disease. In addition, we found a linear dose–response relationship in that a 10 nmol/L increase in 25-hydroxyvitamin D level may lead to a 5% decrease in the risk of dementia (relative risk, 0.95; 95% confidence interval, 0.93–0.98) and 7% in the risk of Alzheimer's disease (relative risk, 0.93; 95% confidence interval, 0.89–0.97).

**Conclusion:** Plasma or serum 25-hydroxyvitamin D concentration was inversely related to the risk of dementia and Alzheimer's disease, consistent with a linear dose–response relationship.

## Introduction

Despite being a common disease, dementia can neither be effectively prevented nor cured at present. With the prolongation of people's lives, the incidence of dementia is also increasing (Abbott, 2011). Nowadays, about 47 million people all over the world suffer from dementia, and the numbers will increase to 131 million by 2050 (Martin et al., 2016). More seriously, the death toll from dementia is high. Alzheimer's disease is the most common type of dementia—death from Alzheimer's disease accounts for 3.4% of all deaths in the United States, ranking it sixth (Kochanek et al., 2015). Therefore, it is of great importance to determine the cause of dementia and spare no effort to prevent it.

Vitamin D is a fat-soluble secosteroid which is generated mainly in our skin by ultraviolet B (UVB) sunlight, and can also be provided by diet (Webb, 2006; Kimlin, 2008; Cashman et al., 2017). Vitamin D is all hydroxylated to 25-hydroxyvitamin D [25(OH)D] in the liver regardless of the source. Thus, 25(OH)D is considered to reflect the vitamin D status in humans (Webb, 2006; Cashman et al., 2017). An increasing number of studies show that vitamin D has a neuroprotective effect (Naveilhan et al., 1996a,b; Brewer et al., 2001; Garcion et al., 2002; Masoumi et al., 2009; Briones and Darwish, 2012). Some cross-sectional studies and subsequent meta-analysis showed that the level of 25(OH)D in patients with Alzheimer's disease is lower than in healthy controls (Balion et al., 2012; Annweiler et al., 2013; Zhao et al., 2013). However, a cross-sectional study cannot determine a causal relationship between low 25(OH)D level and dementia or Alzheimer's disease. Although some cohort studies overcame this problem, their conclusions were not consistent. Some studies showed that low 25(OH)D level increased the risk of dementia and Alzheimer's disease (Annweiler et al., 2011; Afzal et al., 2014; Knekt et al., 2014; Littlejohns et al., 2014; Feart et al., 2017; Licher et al., 2017) but others did not (Graf et al., 2014; Schneider et al., 2014; Karakis et al., 2016; Olsson et al., 2017).

The meta-analysis of Shen and Ji (2015) showed an association between lower vitamin D status and increased risk of dementia and Alzheimer's disease, but a causal relationship cannot be confirmed because it included few cohort studies. Another meta-analysis (Sommer et al., 2017) found a similar result; however, this did not evaluate the risk of Alzheimer's disease. The recent meta-analysis by Jayedi et al. (2018) did not make full use of data because of restricting categories of 25(OH)D level. All previous analyses used the *I*^2^ statistic to describe and quantify the heterogeneity; however, Borenstein et al. (2017) showed that this is not an absolute measure of heterogeneity. Thus, we conducted a random-effects dose–response meta-analysis on cohort studies using prediction interval (PI) to describe the heterogeneity, with the hope of better exploring the relationship between 25(OH)D level and dementia and Alzheimer's disease.

## Materials and methods

### Search strategy

This meta-analysis was carried out according to the PRISMA guidelines and was registered in International Prospective Register of Systematic Reviews (number CRD42018085851; Moher et al., 2009).

We sought cohort studies about the association between the level of vitamin D and the risk of dementia or Alzheimer's disease by searching Pubmed, Embase, Cochrane, and Web of Science databases. Pubmed search terms were: (Alzheimer^*^ OR dement^*^) AND (humans OR persons OR inpatients OR outpatients OR patients OR volunteers OR subjects OR participants) AND (vitamin D OR cholecalciferol OR ergocalciferol OR 25-hydroxyvitamin D) AND (cohort OR cohort studies [Mesh] OR risk [Mesh]). We also carried out manual retrieval using references from literature. No time or language restriction existed during the whole searching process.

### Study selection

Studies included had to meet the following criteria: (1) subjects were humans; (2) exposure was the level of 25(OH)D in plasma or serum; (3) outcome of the study was dementia or Alzheimer's disease and the diagnostic standards were provided; (4) dementia or Alzheimer's disease risk of different 25(OH)D levels was compared; (5) relative risk (RR), hazard ratio (HR) or odds ratio (OR) with 95% confidence intervals (95% CI) was provided; and (6) they were cohort studies. We excluded articles that met the following standards: (1) case–control or cross-sectional study; (2) letter, review, editorial or supplement; and (3) non-human study. If the same study was published repeatedly, we included the one with longer follow-up time.

### Data extraction and quality assessment

Two investigators were independently responsible for data extraction and study quality assessment. Disagreement was solved by asking a third investigator. The following information was extracted from included studies: authors, year of publication, location, study design, follow-up time, categories of 25(OH)D levels, number of participants, number of cases, age, and sex of the participants, adjusted factors, and RRs, HRs or ORs with 95% CI. The quality of studies was assessed based on the Newcastle–Ottawa scale (Wells et al., 2011), and studies with more than six stars were considered to be high quality.

### Statistical analysis

For all studies, we took RRs and 95% CI as effect size, both HRs and ORs were considered to be equivalent to RRs. The study stratified by sex or race was considered as two independent studies. For the study that the unit of 25(OH)D concentration was ng/ml, we multiplied the value to 2.5, converting the units into nmol/L (Holick, 2007; Ross et al., 2011). The 25(OH)D level was divided into different categories in different studies, and we extracted the mean or median 25(OH)D concentration of each category. If the mean or median was not provided, we extracted the midpoint of each category. If the upper boundary of the highest category was not provided, we considered 1.2 times of its lower boundary as the midpoint. If the lower boundary of the lowest category was not provided, we considered it to be 0 nmol/L.

We pooled the RRs for the highest vs. lowest category of 25(OH)D level in different studies using a random-effect model (DerSimonian and Laird, 2015). The publication bias was evaluated by funnel plots, Egger's test as well as Begg's test (Egger et al., 1997). Moreover, we estimated the potential non-linear trends between 25(OH)D level and the risk of dementia and Alzheimer's disease using restricted cubic spline models with three knots (10, 50, and 90%) (Durrleman and Simon, 1989). After calculating the *p*-value for non-linearity (Greenland and Longnecker, 1992), we evaluated the linear trend using the method of generalized least square (Orsini et al., 2012).

The Q-test statistic was chose to evaluate heterogeneity among studies (Higgins et al., 2003), with *p*-values < 0.10 considered to have significant heterogeneity. The *I*^2^ statistic was chose to evaluate the proportion of the variance resulting from heterogeneity in the observed variance and PI was also chosen to describe the heterogeneity (Borenstein et al., 2017).

Finally, we performed a subgroup analysis of the risk of dementia. This analysis was stratified by age, percentage of women, number of participants, number of cases, location, follow-up time, study quality, sample, and whether adjusted for season.

All analyses were conducted using STATA version 12.0 (StataCorp, TX) with a random-effect model. Significant level in this meta-analysis was set as 0.05.

## Results

### Study characteristics

**Figure 1:**
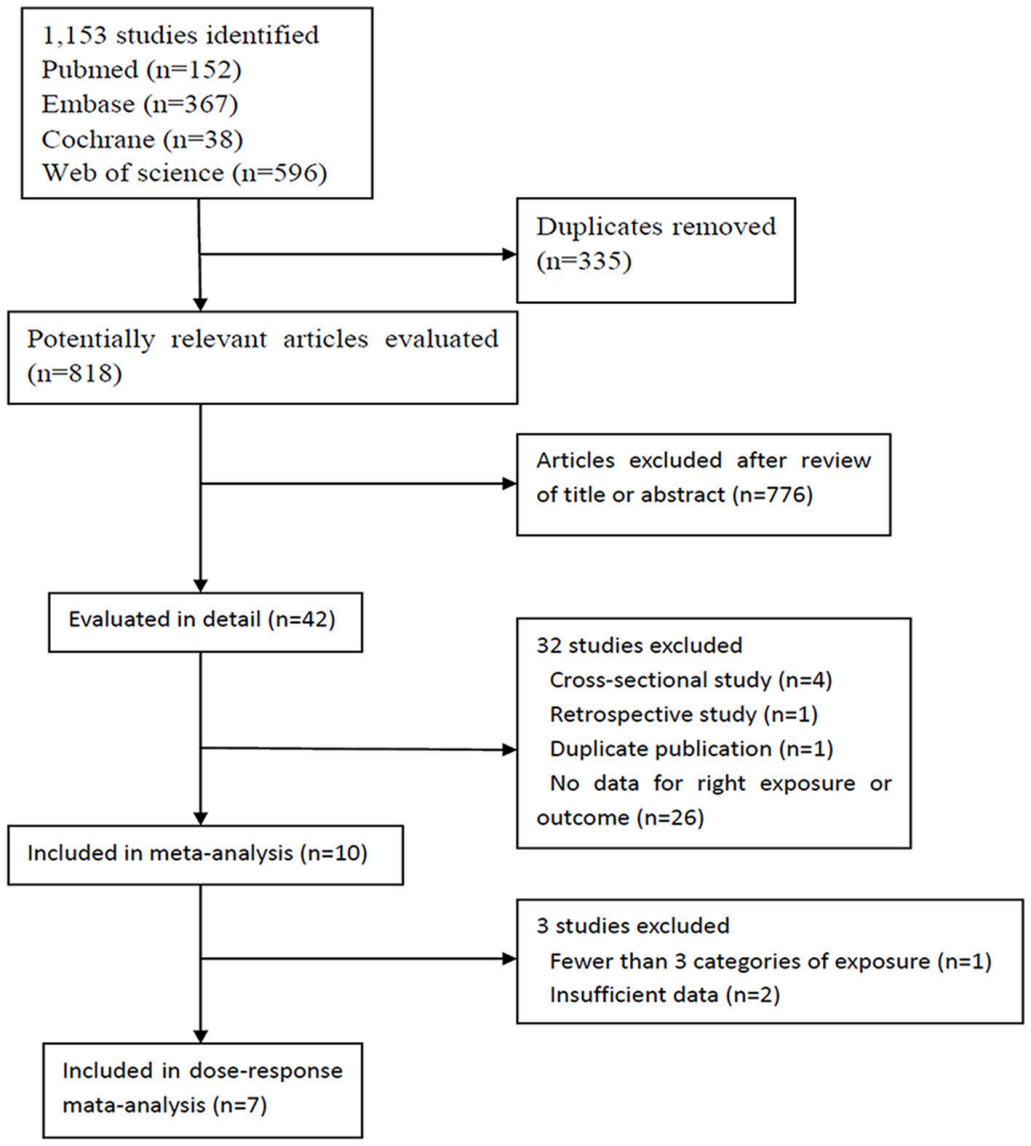
Flow chart of study selection.

Our search identified 1,120 studies, of which 1,110 were excluded from, and ten were included in our meta-analysis (Annweiler et al., 2011; Afzal et al., 2014; Graf et al., 2014; Knekt et al., 2014; Littlejohns et al., 2014; Schneider et al., 2014; Karakis et al., 2016; Feart et al., 2017; Licher et al., 2017; Olsson et al., 2017) (Figure 1). These ten studies were published between 2011 and 2017, and had a total of 28,640 participants; the details of them are shown in Table 1. Three studies (Littlejohns et al., 2014; Schneider et al., 2014; Karakis et al., 2016) were conducted in the USA and seven (Annweiler et al., 2011; Afzal et al., 2014; Graf et al., 2014; Knekt et al., 2014; Feart et al., 2017; Licher et al., 2017; Olsson et al., 2017) in Europe. One study (Annweiler et al., 2011) was carried out for women and another for men (Olsson et al., 2017). In addition, one study (Knekt et al., 2014) reported information of men and women separately, and another (Schneider et al., 2014) reported blacks and whites separately. Because results stratified by sex or race were considered as two independent studies, we finally included 12 studies in our meta-analysis. The result of study quality assessment is shown in Table 2. All these studies were of high quality except one (Annweiler et al., 2011). The measurements of 25(OH)D level, diagnostic criteria for dementia and Alzheimer's disease, and the extracted RRs in these studies are shown in Supplementary Table S1.

**Table 1:** Characteristics of studies included.

**References**	**Location**	**No. of participants**	**Age (years)**	**Percentage of women (%)**	**Follow-up years**	**Adjustments**
Annweiler et al., 2011	France	40	median 78.4	100	7	–
Afzal et al., 2014	Denmark	10186	median 57.6	56	21	Age, sex, month of blood sample, smoking status, BMI, leisure time and work-related activity, income level, alcohol consumption, education, baseline diabetes hypertension, mellitus, cholesterol, creatinine, high-density lipoprotein cholesterol
Graf et al., 2014	Switzerland	246	mean 84.6	76	2	Age, sex, education, basic and instrumental activities, comorbidities, Cumulative Illness Rating Scale, calcemia, Vitamin B12 status, ApoE ε4, mini nutritional assessment, BMI, albuminemia
Knekt et al., 2014	Finland	5010	mean 56	55	17	Age, sex, education, month of blood draw, marital status, smoking, physical activity, alcohol consumption, BMI, plasma fasting glucose, blood pressure, serum total cholesterol, serum triglycerides
Littlejohns et al., 2014	USA	1658	mean 73.6	69	5.6	Age, sex, season, education, smoking, alcohol consumption, BMI, depressive symptoms
Schneider et al., 2014	USA	1652	mean 62.3	60	16.6	Age, sex, education, income, smoking, alcohol use, physical activity, BMI, waist circumference, vitamin D supplementation
Karakis et al., 2016	USA	1663	mean 72.4	59	9	Age, sex, smoking, hypertension, diabetes, homocysteine, prevalent cardiovascular disease, BMI, vitamin D supplementation
Feart et al., 2017	France	916	mean 73.3	62	11.4	Sex, education, income, depressive symptomatology, ApoE ε4, number of drugs per day, BMI, practice of physical exercise, history of stroke and cardiovascular diseases, diabetes, hypertension, hypercholesterolemia, hypertriglyceridemia, Mediterranean diet score, smoking
Licher et al., 2017	Netherlands	6087	mean 69.2	57	13.3	Age, sex, season, BMI, systolic blood pressure, education, diastolic blood pressure, smoking, alcohol use, ethnicity, calcium serum levels, estimated glomerular filtration rate, high-density lipoprotein cholesterol, total cholesterol, history of type 2 diabetes, stroke, heart failure, depressive symptoms, myocardial infarction, outdoor activity, APOE ε4
Olsson et al., 2017	Sweden	1182	mean 71	0	12	Age, season, BMI, education, smoking, physical activity, diabetes, hypertension, hypercholesterolemia, vitamin D supplementation, alcohol intake

*Abbreviations:ApoE ε4, apolipoprotein E ε4; BMI, body mass index*.

**Table 2:** Quality assessment.

**References**	**Selection**				Comparability^*^	**Outcome/exposure**		
	**1**	**2**	**3**	**4**	**1**	**1**	2^**^	**3**
Annweiler et al., 2011	✰	✰	✰	✰		✰	
Afzal et al., 2014	✰	✰	✰	✰	✰✰	✰	✰	✰
Graf et al., 2014		✰	✰	✰	✰✰	✰		✰
Knekt et al., 2014	✰	✰	✰	✰	✰✰	✰	✰	✰
Littlejohns et al., 2014	✰	✰	✰	✰	✰✰	✰		✰
Schneider et al., 2014	✰	✰	✰	✰	✰✰	✰	✰	✰
Karakis et al., 2016	✰	✰	✰	✰	✰✰	✰		
Feart et al., 2017	✰	✰	✰	✰	✰	✰	✰	✰
Licher et al., 2017	✰	✰	✰	✰	✰✰	✰	✰	✰
Olsson et al., 2017	✰	✰	✰	✰	✰✰	✰	✰	✰

* Adjusted variables in different studies are listed in Table 1.

** *Studies with more than ten years of follow-up are marked with a ✰*.

### 25(OH)D level and risk of dementia

**Figure 2:**
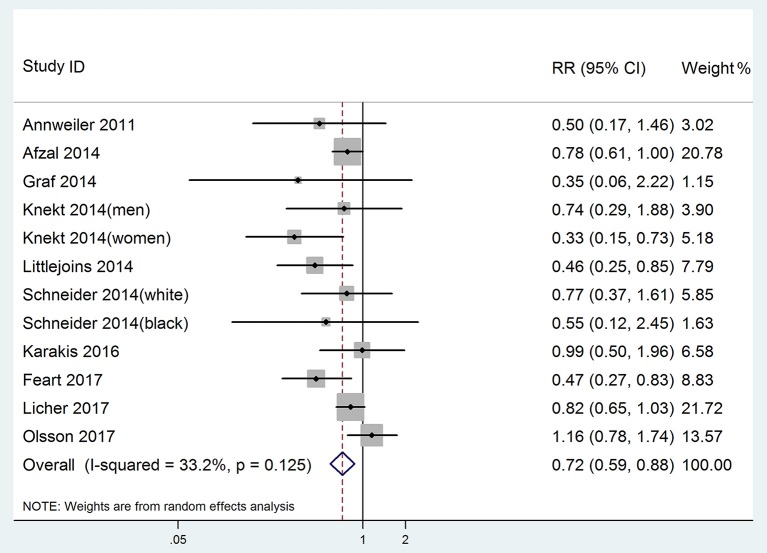
Forest plot of the highest vs. lowest meta-analysis of 25(OH)D level and dementia risk.

All 12 studies were included in highest vs. lowest meta-analysis of the relationship between 25(OH)D level and risk of dementia. In one study (Afzal et al., 2014), the combined Alzheimer's disease and vascular dementia were treated as all-cause dementia. The pooled RR of dementia was 0.72 (95% CI: 0.59–0.88), without significant heterogeneity (*p* = 0.125, *I*^2^ = 33.2%, PI: 0.45–1.14; Figure 2). Egger's test (*p* = 0.102) and Begg's test (*p* = 0.115) showed no significant publication bias. Sensitivity analysis conducted by removing one study at a time and analyzing the rest showed that no single study influenced the pooled RRs (range 0.68–0.77).

**Figure 3:**
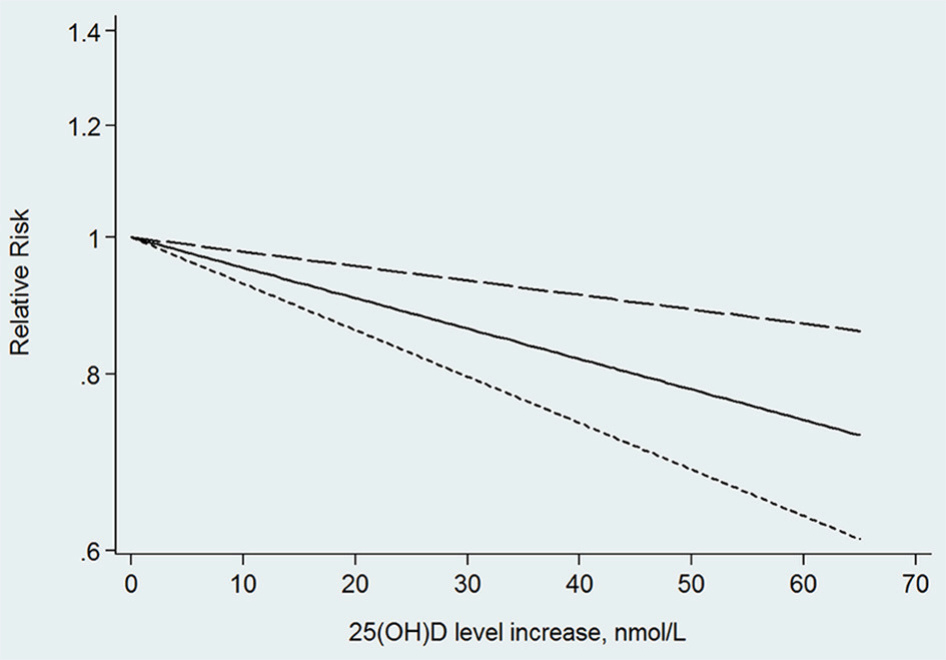
Linear trend of the relationship between 25(OH)D level increase and the risk of dementia.

In the next dose–response meta-analysis, we excluded three of the 12 studies. One of the studies divided the 25(OH)D level into only two categories (Annweiler et al., 2011), and the other two studies did not provide numbers of participants and cases in each category (Graf et al., 2014; Karakis et al., 2016). The non-linear association between 25(OH)D level and the risk of dementia was non-significant (*p* for non-linearity = 0.176). The linear trend showed that the risk of dementia decreased by 5% (RR: 0.95, 95% CI: 0.93–0.98, *p* = 0.000) for every 10 nmol/L increment in 25(OH)D level, without significant heterogeneity (*p* = 0.145; Figure 3).

### 25(OH)D level and risk of alzheimer's disease

**Figure 4:**
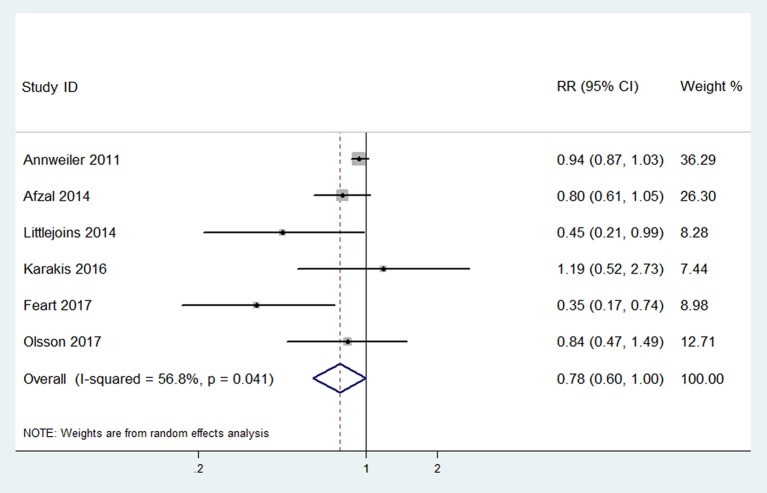
Forest plot of the highest vs. lowest meta-analysis of 25(OH)D level and the risk of Alzheimer's disease.

Six studies (Annweiler et al., 2011; Afzal et al., 2014; Littlejohns et al., 2014; Karakis et al., 2016; Feart et al., 2017; Olsson et al., 2017) with data concerning the risk of Alzheimer's disease were included in the highest vs. lowest meta-analysis. The pooled RR of Alzheimer's disease was 0.78 (95% CI: 0.60–1.00), with significant heterogeneity (*p* = 0.041, *I*^2^ = 56.8%, PI: 0.39–1.54; Figure 4). The RRs after sensitivity analysis were in the range of 0.69–0.88. It is noteworthy that the effect became 0.88 (0.76–1.03) without heterogeneity (*p* = 0.287, *I*^2^ = 20.0%, PI: 0.61–1.31) when we removed one study (Feart et al., 2017) that had not adjusted for age.

**Figure 5:**
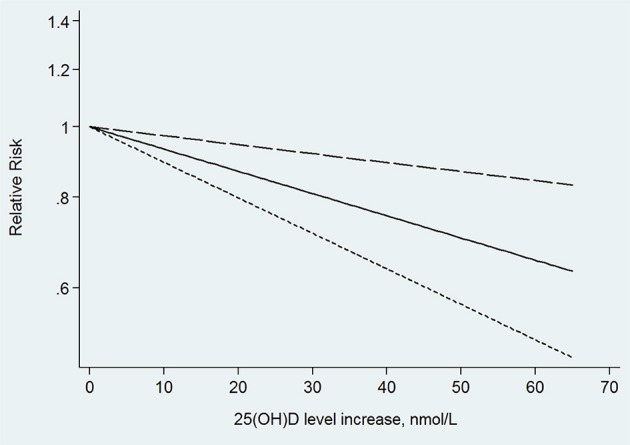
Linear trend of the relationship between 25(OH)D level increase and the risk of Alzheimer's disease.

In the next dose–response meta-analysis, we excluded two (Annweiler et al., 2011; Karakis et al., 2016) of these six studies, because they did not provide enough categories of exposure or numbers of participants and cases in each category. The non-linear association between 25(OH)D level and the risk of Alzheimer's disease was non-significant (*p* for non-linearity = 0.804). The linear trend showed that the risk of Alzheimer's disease decreased by 7% (RR: 0.93, 95% CI: 0.89–0.97, *p* = 0.001) for every 10 nmol/L increment in 25(OH)D level without significant heterogeneity (*p* = 0.200; Figure 5).

### Subgroup analysis

The results of subgroup analysis are shown in Table 3. The associations between 25(OH)D level and the risk of dementia and Alzheimer's disease were consistent in different subgroups, which were defined by age, percentage of women, number of participants, number of cases, location, follow-up time, quality of studies, sample, and whether they were adjusted for season.

**Table 3:** Subgroup analysis of the risk of dementia and Alzheimer's disease.

		**No. of studies**	**RR (95% CI)**	**P for heterogeneity**	***P***
**DEMENTIA**					
1	Age (mean/median)			
	>65	4	0.96 (0.93, 0.99)	0.028	0.017
	≤65	5	0.93 (0.89, 0.97)	0.669	0.002
2	Percentage of women
	>60	4	0.84 (0.78, 0.90)	0.965	0.000
	≤60	5	0.97 (0.94, 1.00)	0.647	0.049
3	No. of participants
	>2000	5	0.97 (0.94, 0.99)	0.187	0.019
	≤2000	4	0.86 (0.81, 0.93)	0.958	0.000
4	No. of cases
	>200	3	0.97 (0.95, 1.00)	0.438	0.069
	≤200	6	0.86 (0.81, 0.91)	0.870	0.000
5	Location
	USA	3	0.87 (0.8, 0.95)	0.950	0.002
	Europe	6	0.96 (0.93, 0.98)	0.100	0.004
6	Follow-up time
	>15	5	0.93 (0.89, 0.97)	0.669	0.002
	≤15	4	0.96 (0.93, 0.99)	0.028	0.017
7	Quality
	>8	7	0.96 (0.94, 0.99)	0.421	0.012
	≤8	2	0.85 (0.78, 0.92)	0.840	0.000
8	Adjusted for Season
	Yes	6	0.96 (0.93, 0.98)	0.077	0.002
	No	3	0.88 (0.80, 0.96)	0.893	0.006
9	Sample
	Serum	6	0.93 (0.90, 0.97)	0.416	0.000
	Plasma	3	0.97 (0.93, 1.00)	0.075	0.087
**ALZHEIMER'S DISEASE**					
1	Age (mean/median)
	>65	3	0.90 (0.85, 0.96)	0.142	0.002
	≤65	1	0.95 (0.90, 1.01)	–	0.096
2	Percentage of women
	>60	2	0.82 (0.74, 0.90)	0.694	0.000
	≤60	2	0.96 (0.95, 1.01)	0.985	0.080
3	No. of participants
	>2000	1	0.95 (0.90, 1.01)	–	0.096
	≤2000	3	0.90 (0.85, 0.96)	0.142	0.002
4	No. of cases
	>200	2	0.96 (0.92, 1.01)	0.985	0.080
	≤200	2	0.82 (0.74, 0.90)	0.694	0.000
5	Location
	USA	1	0.83 (0.73, 0.94)	–	0.004
	Europe	3	0.95 (0.90, 0.99)	0.310	0.013
6	Follow-up time
	>15	1	0.95 (0.90, 1.01)	–	0.096
	≤15	3	0.90 (0.85, 0.96)	0.142	0.002
7	Quality
	>8	2	0.96 (0.92, 1.01)	0.985	0.080
	≤8	2	0.82 (0.74, 0.90)	0.694	0.000
8	Adjusted for season
	Yes	3	0.94 (0.90, 0.99)	0.441	0.009
	No	1	0.80 (0.69, 0.94)	0.276	0.006
9	Sample			
	Serum	1	0.83 (0.73, 0.94)	0.669	0.004
	Plasma	3	0.95 (0.90, 0.99)	0.310	0.013

## Discussion

In our meta-analysis, 25(OH)D level was inversely associated with the risk of dementia and Alzheimer's disease, and the subgroup analysis indicated a similar result. In addition, we found a linear dose–response relationship showing that a 10 nmol/L increase in 25(OH)D level may lead to a 5% decrease in dementia and 7% in Alzheimer's disease.

A previous meta-analysis (Shen and Ji, 2015) that included only three cross-sectional studies and two prospective cohort studies was not regarded as convincing evidence because there were too few participants, and was inadequate to determine any causal relationship between low 25(OH)D level and dementia or Alzheimer's disease. Two years later, a meta-analysis (Sommer et al., 2017) of five cohort studies showed that low 25(OH)D level may lead to a high risk of dementia. However, this analysis included studies published only in 2014. After that, four studies (Karakis et al., 2016; Feart et al., 2017; Licher et al., 2017; Olsson et al., 2017) of high quality appeared, and two (Karakis et al., 2016; Olsson et al., 2017) found no association between 25(OH)D level and dementia or Alzheimer's disease. A more recent meta-analysis (Jayedi et al., 2018) included only studies with more than three categories of 25(OH)D level and excluded information from studies with fewer categories of 25(OH)D level. Our meta-analysis included 28,640 participants and quantified the relationship between 25(OH)D level and dementia or Alzheimer's disease and provided a clearer understanding about 25(OH)D level and the risk of dementia or Alzheimer's disease.

Several mechanisms may explain the relationship between vitamin D and dementia and Alzheimer's disease. Vitamin D can play a neuroprotective role by up-regulating nerve growth factor, brain-derived neurotrophic factor and glial cell-derived neurotrophic factor (Naveilhan et al., 1996a,b; Garcion et al., 2002). Both animal and human studies have found that vitamin D can promote the removal of amyloid-β (Aβ; Masoumi et al., 2009; Briones and Darwish, 2012; Mizwicki et al., 2012). Vitamin D can also inhibit the expression of inducible nitric oxide synthase (Dursun et al., 2013). Furthermore, vitamin-D-binding protein can reduce Aβ aggregation and cell death induced by Aβ (Moon et al., 2013).

There are some strengths in our study. On the one hand, we included only cohort studies in our meta-analysis, and most were of high quality. Thus, selection bias and reverse causality were largely reduced. On the other hand, unlike previous meta-analyses, we assessed the relationship between 25(OH)D level and dementia or Alzheimer's disease with a dose–response model which enabled a better use of information. Moreover, we used PI to evaluate the heterogeneity in this random-effects meta-analysis. The PI ranged within 0.45–1.14 for risk of dementia and 0.39–1.54 for risk of Alzheimer's disease. Thus, it is possible that future studies will show an increased dementia and Alzheimer's disease risk in people with higher compared with lower 25(OH)D level.

However, our meta-analysis has several limitations. Firstly, we focused on the relationship between baseline 25(OH)D levels and the risk of dementia and Alzheimer's disease. It was unclear whether the 25(OH)D level had changed during the follow-up. However, this effect is likely to be small because studies have shown that 25(OH)D concentration can be relatively stable for a long period (Jorde et al., 2010; Sonderman et al., 2012). Secondly, all participants involved in our meta-analysis were Americans and Europeans, thus our findings may not apply to people elsewhere in the world. Thirdly, in studies included in our meta-analysis, the diagnostic criteria for dementia and Alzheimer's disease as well as measurements of 25(OH)D concentration were not completely unified and this may lead to some bias. However, diagnostic criteria such as NINCDS-ADRDA, DSM-IV, DSM-III, ICD-8, ICD-9, and ICD-10 are all well accepted and the measurements of 25(OH)D concentration were accurate. Considering the different test samples (plasma and serum) in these studies, we conducted a subgroup analysis and found similar results. Fourthly, there are several subtypes of dementia, such as Alzheimer's disease, vascular dementia and dementia with Lewy bodies. In our meta-analysis, the only dementia subtype was Alzheimer's disease, which is the most common subtype. The relationship between 25(OH)D and other subtypes of dementia remains to be discussed. Error in distinguishing different types of dementia may result in attenuation of the true association between 25(OH)D level and Alzheimer's disease. Moreover, the confounding factors adjusted in each study differed, and the risk factors for dementia were still not clear. Accordingly, there may be some risk factors to be adjusted. In this meta-analysis, we extracted RRs with most adjusted factors, and the study (Annweiler et al., 2011) without any adjusted factors was excluded in the dose–response analysis because of its categories of exposure. Lastly, due to the observational design of all the studies, the possibility of potential bias and residual confounding cannot be excluded.

Finally, because we found that a higher level of 25(OH)D could help to prevent dementia and Alzheimer's disease, ways to increase 25(OH)D level should be given attention. Approximately 80–100% of the vitamin D required by humans is produced by the UVB radiation in sunshine (Kimlin, 2008). However, at higher latitudes, UVB availability decreases and the number of months in which vitamin D cannot be effectively synthesized increases (O'Neill et al., 2016). Nevertheless, this does not mean that people in higher latitudes have a higher prevalence of vitamin D deficiency because diet, clothing, skin color as well as behavior habits also influence synthesis of vitamin D (Jablonski and Chaplin, 2012; O'Neill et al., 2016). Having more outdoor activities and eating more food rich in vitamin D such as fish (cod liver), meat, eggs and milk (Ovesen et al., 2003; Brustad et al., 2004; Hayes and Cashman, 2017) may help to improve vitamin D status. In addition, vitamin D supplementation and some vitamin D-fortified food can also improve vitamin D intake (O'Donnell et al., 2008; Black et al., 2012; Cashman and Kiely, 2016; Manios et al., 2017). Moreover, it is important to note that the safe upper intake level for vitamin D is 100 μg/day for adults, and long-term intake of too much vitamin D can be harmful (IOM, 2011; EFSA, 2012).

## Conclusion

In summary, this dose–response meta-analysis showed that plasma or serum 25(OH)D level was inversely associated with the risk of dementia and Alzheimer's disease, consistent with a linear dose–response relationship. Maintaining adequate 25(OH)D status may lower risk of dementia and Alzheimer's disease.

## Author contributions

HC and WT designed the study. HC and LT searched the databases. WT and WX selected studies. JL and KF evaluated the quality of studies. HC and WT performed all analyses. HS and BZ wrote the manuscript. All authors revised the manuscript.

### Conflict of interest statement

The authors declare that the research was conducted in the absence of any commercial or financial relationships that could be construed as a potential conflict of interest.
