# Supplementary material for: 25-Hydroxyvitamin D Levels and the Risk of Dementia and Alzheimer's Disease: A Dose–Response Meta-Analysis
Source: Front Aging Neurosci. 2018 Nov 9;10:368. doi: 10.3389/fnagi.2018.00368 (PMC6237859; doi:10.3389/fnagi.2018.00368)
Supplement: Supplementary file 1 [file Table_1.DOCX]

Supplementary Material

**25-Hydroxyvitamin D Levels and the Risk of Dementia and Alzheimer’s Disease: A Dose-Response Meta-Analysis**

***Hanze Chen1, Weishuang Xue1, Jinwei Li1, Kailei Fu1, Han Shi2, Beidi Zhang3, Weiyu Teng1*and Li Tian4****

****Correspondence:*** *Dr. Weiyu Teng****:*** [*tengweiyucmu@sina.com*](http://mailto:tianlicmu2%40sina%2Ecom/)*; Dr. Li Tian****:*** [*tianlicmu2@126.com*](http://mailto:tianlicmu2@126.com/)

**Supplementary Table S1︱**Details of exposure and outcome and extracted RRs (95% CI)

| Author | Exposure and measurement | Outcome and ascertainment | Categories of 25(OH)D | RR (95% CI) |
| --- | --- | --- | --- | --- |
| (Annweiler et al., 2011) | Serum 25(OH)D level: Radioimmunoassay | Dementia: DSM-IV  Alzheimer’s disease: NINCDS-ADRDA | Dementia:  <10 ng/ml  ≥10 ng/ml  Alzheimer’s disease:  <10 ng/ml  ≥10 ng/ml | 2.02 (0.69–5.94)  Reference  1.06 (0.97–1.15)  Reference |
| (Afzal et al., 2014) | Plasma 25(OH)D level: DiaSorin Liaison 25(OH)D TOTAL assay | Alzheimer’s disease: ICD-8/10, national Danish Patient Registry and the national Danish Causes of Death Registry | Dementia:  <25 nmol/L  25–49.9 nmol/L  ≥50 nmol/L  Alzheimer’s disease:  <25 nmol/L  25–49.9 nmol/L  ≥50 nmol/L | 1.28 (1.00–1.64)  1.16 (0.94–1.42)  Reference  1.25 (0.95–1.64)  1.12 (0.90–1.40)  Reference |
| (Graf et al., 2014) | Plasma 25(OH)D level: electrochemiluminesence immunoassay | Dementia: DSM IV-TR, NINCDS-ADRDA, ADDTC and NINDS-AIREN | Dementia:  <25 nmol/L  25–50 nmol/L  50–75 nmol/L  ≥75 nmol/L | 2.85 (0.45–17.95)  6.18 (0.87–43.76)  2.87 (0.36–22.77)  Reference |
| (Knekt et al., 2014) | Serum 25(OH)D level: radioimmunoassay | Dementia: ICD-8 and nationwide Finnish Hospital Discharge Register | Dementia:  Male:  7–28 nmol/L  29–39 nmol/L  40–53 nmol/L  54–159 nmol/L  Female:  7–28 nmol/L  29–39 nmol/L  40–53 nmol/L  54–159 nmol/L | Reference  0.41 (0.14–1.19)  0.63 (0.25–1.56)  0.74 (0.29–1.88)  Reference  0.90 (0.56–1.44)  0.60 (0.34–1.06)  0.33 (0.15–0.73) |
| (Littlejohns et al., 2014) | Serum 25(OH)D level: liquid chromatography-tandem mass spectrometry | Dementia and Alzheimer’s disease: NINCDS-ADRDA | Dementia:  <25 nmol/L  25–49.9 nmol/L  ≥50 nmol/L  Alzheimer’s disease:  <25 nmol/L  25–49.9 nmol/L  ≥50 nmol/L | 2.18 (1.18–4.02)  1.50 (1.04–2.17)  Reference  2.20 (1.01–4.80)  1.65 (1.03–2.65)  Reference |
| (Schneider et al., 2014) | Serum 25(OH)D level: liquid chromatography-tandem mass spectrometry | Dementia: ICD-9, hospital discharge record | Dementia:  White:  <20 ng/ml  20–<30 ng/ml  ≥30 ng/ml  Black:  <20 ng/ml  20–<30 ng/ml  ≥30 ng/ml | 1.30 (0.62–2.71)  1.29 (0.70–2.40)  Reference  1.81 (0.33–6.50)  1.47 (0.42–7.82)  Reference |
| (Karakis et al., 2016) | Serum 25(OH)D level: radioimmunoassay | Dementia: DSM-IV  Alzheimer’s disease: NINCDS-ADRDA | Dementia:  <12 ng/ml  12–<20 ng/ml  20–<50 ng/ml  Alzheimer’s disease:  <12 ng/ml  12–<20 ng/ml  20–<50 ng/ml | 1.00 (0.58–1.72)  0.90 (0.65–1.23)  Reference  0.72 (0.37–1.42)  0.76 (0.52–1.11)  Reference |
| (Feart et al., 2017) | Plasma 25(OH)D level: one-step immunoassay | Dementia and Alzheimer’s disease: DSM-IV and NINCDS-ADRDA | Dementia:  <25 nmol/L  25–50 nmol/L  >50 nmol/L  Alzheimer’s disease:  <25 nmol/L  25–50 nmol/L  >50 nmol/L | 2.12 (1.21–3.71)  1.98 (1.17–3.36)  Reference  2.85 (1.36–5.97)  2.78 (1.37–5.68)  Reference |
| (Licher et al., 2017) | Serum 25(OH)D level: electrochemiluminescence binding assay | Dementia: DSM-III-R  Alzheimer’s disease: NINCDS-ADRDA | Dementia:  <25 nmol/L  25–<50 nmol/L  ≥50 nmol/L | 1.22 (0.97–1.52)  1.06 (0.90–1.26)  Reference |
| (Olsson et al., 2017) | Plasma 25(OH)D level: HPLC–mass spectrometry | Dementia and Alzheimer’s disease: medical records, DSM-IV and NINCDS-ADRDA | Dementia:  ≤50 nmol/L  >50–75 nmol/L  >75 nmol/L  Alzheimer’s disease:  ≤50 nmol/L  >50–75 nmol/L  >75 nmol/L | 0.86 (0.58–1.30)  0.90 (0.68–1.19)  Reference  1.19 (0.67–2.12)  1.11 (0.73–1.67)  Reference |

Abbreviations: DSM-IV, Diagnostic and Statistical Manual of Mental Disorders 4th Edition; DSM IV-TR, Diagnostic and Statistical Manual of Mental Disorders, edition 4 text revision; NINCDS-ADRDA, National Institute of Neurological and Communication Disorders and Stroke and Alzheimer’s Disease and Related Disorders Association; ICD, International Classification of Diseases; ADDTC, Alzheimer’s Disease Diagnostic and Treatment Centres; NINDS-AIREN, National Institute of Neurological Disorders and Stroke and Association Internationale pour la Recherché et l’Enseignement en Neurosciences; DSM-III-R, [Diagnostic and Statistical Manual of Mental Disorders, Third Edition, Revised](http://xueshu.baidu.com/s?wd=paperuri%3A(6600f499e4ddfcaa0118acfd655cae4b)&filter=sc_long_sign&tn=SE_baiduxueshu_c1gjeupa&ie=utf-8&sc_ks_para=q%3DDiagnostic%20and%20Statistical%20Manual%20of%20Mental%20Disorders%2C%20Third%20Edition%2C%20Revised); HPLC–mass spectrometry, HPLC atmospheric pressure chemical ionization-mass spectrometry.
